# Resting-State Functional Connectivity in Autism Spectrum Disorders: A Review

**DOI:** 10.3389/fpsyt.2016.00205

**Published:** 2017-01-04

**Authors:** Jocelyn V. Hull, Lisa B. Dokovna, Zachary J. Jacokes, Carinna M. Torgerson, Andrei Irimia, John Darrell Van Horn

**Affiliations:** ^1^Laboratory of Neuro Imaging (LONI), The Institute for Neuroimaging and Informatics (INI), Keck School of Medicine of USC, University of Southern California, Los Angeles, CA, USA

**Keywords:** resting state, fMRI, functional connectivity, autism spectrum disorder, developmental brain imaging, neural networks

## Abstract

Ongoing debate exists within the resting-state functional MRI (fMRI) literature over how intrinsic connectivity is altered in the autistic brain, with reports of general over-connectivity, under-connectivity, and/or a combination of both. Classifying autism using brain connectivity is complicated by the heterogeneous nature of the condition, allowing for the possibility of widely variable connectivity patterns among individuals with the disorder. Further differences in reported results may be attributable to the age and sex of participants included, designs of the resting-state scan, and to the analysis technique used to evaluate the data. This review systematically examines the resting-state fMRI autism literature to date and compares studies in an attempt to draw overall conclusions that are presently challenging. We also propose future direction for rs-fMRI use to categorize individuals with autism spectrum disorder, serve as a possible diagnostic tool, and best utilize data-sharing initiatives.

## Introduction

Autism spectrum disorder (ASD) encompasses a range of developmental disorders characterized by deficits in social communication and interaction and restricted and repetitive behaviors (1). Individuals with ASD are found along an entire continuum of cognitive abilities, ranging from profound disability to exceptional intelligence (2). “Early infantile autism” was first recognized in 1943 by Kanner (3), although it was not until 1980 that “infantile autism” was added to the third edition of the Diagnostic and Statistical Manual of Mental Disorders (DSM-III). The name was later changed to “autistic disorder” in 1987 (4). Asperger’s Syndrome was added to DSM-IV in 1994, 50 years after Hans Asperger’s observations (4). Under the DSM-V edition, autism, Asperger’s, and pervasive developmental disorder-not otherwise specified are grouped together as part of a broader diagnosis known as ASDs. This new classification acknowledges both the range and severity of impairments seen in ASD. The spectrum approach thus addresses the inherently heterogeneous nature of autism and attempts to better represent the variability of phenotypes found in the disorder.

The Autism and Developmental Disabilities Monitoring Network, established by the CDC to determine autism prevalence in the US, has reported markedly increasing prevalence with rates among 8-year-old children of 1 in 110, 1 in 88, and 1 in 68 for 2006, 2008, and 2010, respectively (5). While the most recent estimate remains at 1 in 68 children, there is still great disparity in diagnosis-by-sex, with males 4.5 times as likely as their female counterparts to be diagnosed with ASD (6). The underlying cause of ASD’s increasing prevalence is contentiously debated with suggestions of both environmental and genetic contributions. Other contributing factors include changes to diagnostic and clinical criteria, greater efficacy of screening methods, and increased recognition of the disorder by parents and the medical community (7).

The introduction of neuroimaging research has provided a means to explore the neurophysiological underpinnings of the disorder. Early neuroimaging studies employed positron emission topography (PET) to examine metabolic changes (8), electroencephalography and magnetoencephalography to observe functional fluctuations in brain activity (9–11), and magnetic resonance imaging (MRI) to investigate structural alterations in ASD (12). Functional MRI (fMRI) is a now commonly used technique to observe functional variations in brain activity during performance of a task by measuring changes in the blood-oxygen level-dependent contrast images (13, 14). The discovery that intrinsic synchronous activity occurs in distant regions of the brain at rest, corresponding to a functional network, led to development of resting-state functional connectivity research in ASD (15). However, the literature would benefit from review and synthesis as conflicting reports of connectivity patterns, either on a local or global level, remain unresolved.

The multitude of neuroimaging techniques also provides a means to test cognitive theories of autism to determine if there is a neurobiological basis for validity. While selected constructs like notions such as the “refrigerator mom theory” have been discredited, maternal affect and interpersonal relationships continue to be examined (16). Other cognitive constructs such as there being a “deficit in theory of mind hypothesis” remain as active areas of research framed in the context of functional imaging examinations.

However, considerable interest now exists in the characterization of brain network-level alterations in patients having ASD as compared to typically developing (TD) children and how differences in putative information transfer might underlie the cognitive and behavioral differences observed. As such, this review will address the cognitive theories which have prompted investigation of the underlying neural causes of ASD, highlight the main findings of ASD rs-fMRI literature to date, acknowledge marked differences in results, and discuss the likely study limitations contributing to these differences. In so doing, we make note of a broad range of resting-state functional connectivity literature. We specifically aimed to be as inclusive as possible but to avoid studies which might have formed re-analyses of a primary dataset where it was obvious. We aimed to include the range of neuroimaging studies examining ASD and TD participants across the critical time period of development from childhood to early puberty. We also sought to include studies which provided sufficient details on their functional imaging data acquisition methodology. Finally, we wished to capture the range of methods for the determination and quantification of functional connectivity including seed voxel-based approaches, multivariate methods, graph theoretical, and other approaches—do so under the thinking that such methods may be contributing to the reports of both over- and under-connectivity characterizing the brain of patients with ASD in contrast to TD individuals. While unlikely to have completely identified every such peer-reviewed study, we believe that our extensive efforts have provided us with a unique perspective on the status of this literature. Moreover, in what follows, we are able to draw useful conclusions about the course forward for obtaining a more comprehensive understanding of the brain’s connectivity in ASD.

## Cognitive Theories of Autism

The majority of scientific literature was initially devoted to describing the type and extent of behavioral dysfunction seen in ASD. Results from this extensive work prompted researchers to develop cognitive theories, many of which emerged in the late 1980s and early 1990s.

The Theory of Mind (ToM) hypothesis, proposed by Baron-Cohen, has emerges as a highly regarded explanation of autistic behavior (17–21). ToM refers to a person’s ability to understand the subjective mental states of others, including their perspective and intentions, whether a scenario is real or hypothetical (22). The ToM hypothesis suggests that individuals with ASD have an absent or underdeveloped ToM which impedes them from inferring the mental states of others—a task that lies at the heart of social interaction (23). Several studies have explored the extent of ToM impairment in ASD (21, 24) and used neuroimaging to investigate a potential neural basis for the theory (25, 26). Baron-Cohen secondarily introduced the Empathizing–Systemizing theory, alternatively called the Extreme Male Brain hypothesis (27–29). In one study, typically developing adult males were more inclined to systemize while females were more likely to empathize. It was then hypothesized that autism is marked by an extreme systemizing approach above and beyond the normal male’s predisposition to systemization (27–29). Social deficits observed in ASD as well as the increased prevalence in males could be explained by this shift along the empathizing–systemizing continuum. Ozonoff and colleagues have proposed the executive dysfunction hypothesis, which suggests that deficits observed in ASDs are the result of poor executive function including working memory, inhibition, mental flexibility, and planning (24). Proponents often pair executive dysfunction theory with ToM as successful communication requires continual analysis of unfolding events and thoughtful response as well as identification of another person’s mental state (30). The Weak Central Coherence theory of autism proposes that behavioral and cognitive symptoms could be explained by the inability to “see the big picture” (31–33). The theory provides a plausible explanation for the improved capabilities in mathematics and engineering seen in ASD individuals, in which it is better to focus on parts rather than the whole (31). Weaker performance on visuospatial, perceptual, and verbal-semantic tasks, all of which require strong central coherence (34–37), support this theory.

Evidence for dysfunction in the mirror neuron system (MNS) is increasingly being implicated in ASD (38–40). Mirror neurons are activated when an individual performs a task or observes someone else performing the same task (22). For this reason, the MNS is the likely neural foundation of imitative behaviors, making it crucial for language learning, perception of social behaviors, and empathy (41, 42). Evidence for this theory is supplied by behavioral fMRI imaging studies involving social expression imitation tasks (41) and examination of rs-fMRI functional connectivity studies that explore the connectivity of brain regions implicated in the MNS (38).

The Weak Central Coherence theory, Executive Dysfunction hypothesis, and deficits in ToM are the dominant cognitive theories still heavily explored in the field (39, 43). Cognitive theories have an important role in spurring alternative avenues of research. The use of neuroimaging techniques to uncover neural origins of cognitive theories has led to the development of further neuroimaging-based theories such as under- and over-connectivity and MNS dysfunction. This highlights the important interplay between cognitive theories informing study direction and neuroimaging providing much-needed revisions to existing theories or, just as importantly, evidence for development of new ones.

## Autism: A Question of Connectivity?

The increasing use of functional connectivity modeling has driven the formation of two primary theories: the brains of patients with ASD expressing under-connectivity or, conversely, showing evidence of over-connectivity (OC). The under-connectivity theory, derived from task-based neuroimaging, suggests that behavioral features of ASD stem from reduced inter-regional neural connections in the brain, particularly in networks that rely on frontal and posterior integration (44, 45). By contrast, OC has been reported between brain networks in subjects with ASD, while more recent evidence implies overall altered connectivity of the brain with combined instances of both over- and under-connectivity.

**Figure 1:**
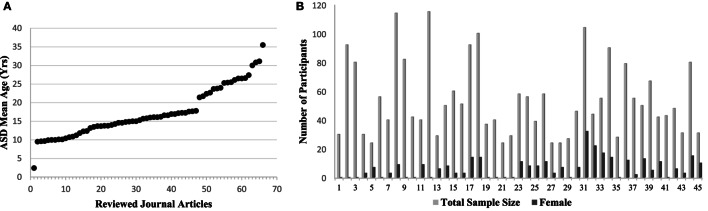
**(A)** The mean age of autism spectrum disorder (ASD) participants per study included in this rs-fMRI review (Table 1). Articles are ordered by increasing age of the study population to emphasize existing trends. Gaps can be seen in age representation from 3 to 9 years and 17 to 21 years, and again a lack of representation in the older age groups. **(B)** Representation of females in each study represented by dark gray bars out of total sample size represented by the light gray bar. Studies are ordered alphabetically according to Table 1. An obvious trend of underrepresentation of females with ASD research can be seen, with increasing sample size not contributing to an increase in females represented. Studies using the Autism Brain Imaging Data Exchange sample set are not shown, but replicate this overall finding.

**Figure 2:**
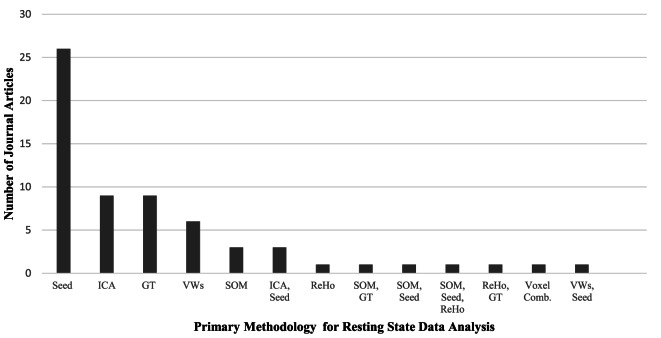
**A comparison of the primary methodology used for resting-state functional MRI data analysis in the autism literature and the number of journal articles that represent each analysis technique**. Abbreviations: Seed, voxel-based-seed analysis; ICA, independent component analysis; GT, graph theory; VWs, voxel-wise whole brain analyses; SOM, self-organizing maps; ReHo, regional heterogeneity; Voxel Comb, voxel combinations. The use of multiple techniques is indicated with two or more abbreviations.

Resting-state fMRI has provided a convenient tool to examine the changes in the intrinsic connectivity of specific regions and networks in ASD and non-ASD subjects. Since the first publications using rs-fMRI in ASD research (46–48), there has been tremendous utilization of this technique, particularly with the introduction of data-sharing initiatives like the Autism Brain Imaging Data Exchange (ABIDE) network (49). Although this upsurge of interest in ASD research is positive, it comes with the formidable challenge of explaining contradictory reports of ASD connectivity. Age and sex representation (Figures 1A,B), methodological considerations (Figure 2), and the heterogeneous nature of ASD could be driving the discrepancies in the literature.

Some clarity is necessary in order to properly contextualize the terms under- and over-connectivity. In what follows, we will then address the studies that reported under-connectivity, followed by those that demonstrated OC, and conclude with studies that found evidence of both types. The reader is referred to Table 1 for a summary of all resting-state FC articles included in this review.

**Table 1:** **Citations included in resting-state functional connectivity functional MRI (fMRI) in autism literature analysis**.

Citation	Method	Connectivity	Global/local	Data set	Scan time (s)	Total sample size	Sex (♀ out of total)	Autism spectrum disorder (ASD) mean age (years)	Brain regions examined
Abrams et al. (50)	Seed	UC	Global	Autism Brain Imaging Data Exchange (ABIDE)	360	39	8	9.96	pSTS
Alaerts et al. (51)	ReHo, Seed	B	Global/local	ABIDE		215	0	16	pSTS
Alaerts et al. (52)	Seed	UC	Global		420	30	0	21.7	Whole brain (240 volumes)
Anderson et al. (53)	SOM	B	Global/local		480	92	0	22.4	
Anderson et al. (54)	GT	OC	Global	ABIDE	Variable	964	142	na	
Anderson et al. (55)	SOM	UC	Global		480	80	0	22.7	Whole brain (90 subdivisions)
Assaf et al. (56)	ICA	UC	Global		315	30	3	15.7	DMN
Barttfeld et al. (57)	Seed	B	Global		442	24	7	23.7	Networks: frontoparietal, cingulo-opercular, DMN, occipital, sensorimotor, cerebellar
Bos et al. (58)	ICA	B	Global/local		600	56	0	11.8	
Cardinale et al. (59)	ICA	B	Global		376	40	3	14.6	Whole brain (networks: frontoparietal, visual, executive control, auditory, sensorimotor)
Cerliani et al. (60)	ICA	OC	Global	ABIDE	na	359	0	17.6	19 RSNs (sensory, FTP, subcortical, cerebellum, paralimbic, saliency, DMN)
Cheng et al. (61)	VWs	B	Global	ABIDE	Variable	927	136	17.17	Whole brain
Cherkassky et al. (46)	Seed	UC	Global		Block	114	9	24	PCC, vACC, PrC, mPFC, PHG, insula, IPC
Chien et al. (62)	Seed	OC	na		360	82	0	12.38	pTPJ
Delmonte et al. (63)	Seed	OC	Global		438	42	0	17.28	IFGpo, IGFpt, frontal pole, MFG, SFG, subcallosal cortex, ACC, precentral gyrus, supplemental motor area, amygdala, putamen, caudate, NACC, paracingulate gyrus
Di Martino et al. (49)	ReHo, Seed, SOM	B	Global/local	ABIDE	Variable	709	0	na	Whole Brain and region-of-interest (ROI) seed (mPFC, PCC)
Di Martino et al. (64)	Seed	OC	Global		398	40	9	10.4	Whole brain (caudate and putamen)
Di Martino et al. (49)	GT	OC	Global	ABIDE	360	149	28	10.1	Whole brain
Dinstein et al. (65)	Seed	UC	Global		Block	72	na	2.42	LPFC, IFG, CS, aIPS, STG, LOS
Doyle-Thomas et al. (66)	Seed	B	Global/local		283	115	0	12.3	DMN (mPFC, ACC, MTG, PrC)
Ebisch et al. (67)	Seed	UC	Global		516	29	6	15.79	Insula, intraparietal sulcus, precentral sulcus, middle temporal regions
Eilam-Stock et al. (68)	VWs, Seed	B	Global/local		360	32	na	26.1	Whole brain and ROI seed (PCC)
Fishman et al. (38)	Seed	B	Global		370	50	8	14.8	ToM (mPFC, TPJ, PCC) and MNS (bilateral aIPS, pSTS, PMC)
Gotts et al. (69)	na				60	3	16.92		
Gotts et al. (70)	VWs	UC	Global		490	51	3	16.92	Whole brain
Hahamy et al. (71)	VWs	B	Global/local	ABIDE	Variable	141	20	26.6	Whole brain
Iidaka (72)	VWs	B	Global/local	ABIDE	Variable	640	100	13.2	
Itahashi et al. (73)	GT	B	Global		na	92	14	31.11	Whole brain
Itahashi et al. (74)	GT	B	Local		na	100	14	30.82	Whole brain
Jones et al. (75)	SOM, Seed	UC			Block	37	0	16.1	
Jung et al. (76)	Seed	UC	Global		462	40	0	25.3	aMPFC and PCC
Kennedy and Courchesne (47)	Seed	UC	Global		430	24	0	26.5	DMN, dorsal attention
Kennedy et al. (48)	Seed	na	na		Block	29	0	25.49	DMN
Keown et al. (77)	GT	B	Local		370	58	11	13.8	Whole brain
Khan et al. (78)	Seed	B	Global		370	56	8	14.27	PFC, S1, STC, premotor and primary motor cortices, PMC, PPC, OOC, inferior MTG, cerebellum
Lee et al. (79)	VWs	UC	Both	ABIDE	Variable	975	144	16.2	Whole brain
Long et al. (80)	Voxel combinations	UC	Both	ABIDE	na	128	20	9.6, 13.7, 25.4^a^	Whole brain
Lynch et al. (81)	Seed	B	Global		360	39	8	9.96	PCC, RsC, PrC, PMC
Maximo et al. (82)	ReHo, GT	B	Local		370	58	11	13.8	Whole brain
Monk et al. (83)	Seed	B	Both		600	24	3	26.5	DMN
Müeller et al. (84)	ICA	UC	na		360	24	7	35.5	Whole brain
Murdaugh et al. (85)	VWs	UC	Global		Block	27	0	21.4	DMN (mPFC, vACC, PCC, PrC, AG, IPL)
Nair et al. (86)	–	B	na		370	46	7	15	
Nebel et al. (87)	Seed	B	Local	ABIDE	Variable	868	0	16.1	Precentral gyrus
Nebel et al. (88)	Seed	O	Local		420	104	32	10.7	Primary motor cortex
Nielson et al. (89)	GT	na		ABIDE	Variable	1,112	142	16.6	Whole brain (7,266 ROIs)
Nomi and Uddin (90)	ICA	B	Global/local	ABIDE	360	144	27	9.51	Whole brain
Olivito et al. (91)	Seed	B	Global		440	44	22	23.8	Whole brain (dentate nucleus seed region)
Paakki et al. (92)	ReHo	B	Local		456	55	17	14.58	Whole brain
Padmanabhan et al. (93)	Seed	B	Global		na	90	14	17.28	Striatum, DC, ventral striatum, putamen
Plitt et al. (94)	Seed	na	na	ABIDE	490	118	0	17.66	
Price et al. (95)	ICA	na	na		360	60	na	9.69	ICN
Ray et al. (96)	GT	OC	Global	ABIDE	300	56	17	10.1	219 Cortical ROIs
Redcay et al. (97)	GT	OC	Global		na	28	0	17.8	Networks: cingulo-opercular, cerebellar, frontoparietal, DMN
Rudie and Dapretto (98)	GT	B	Global/local		360	79	12	13.5	Networks: visual, motor, attention, DMN
Shukla et al. (99)	–	B	Local		Block	55	2	13.7	
Starck et al. (100)	ICA	UC	Global/local		450	50	13	14.9	DMN
Superkar et al. (101)	ICA	Default mode network development in typically developing children		44		24			
Tyszka et al. (102)	ICA, Seed	UC	na		300	39	na	27.4	Whole brain
Uddin et al. (103)	Seed	B	Global/local		360	67	5	9.9	Networks: CEN (r-dlPFC, rPPC) and SN (right frontal insula, ACC)
Verly et al. (104)	Seed	UC	Global		420	42	11	14	IFG, STG, dlPFC, MFG, premotor cortex, cerebellar lobule VI, Crus I
von dem Hagen et al. (105)	ICA, Seed	UC	Global		598	43	0	30	mPFC, PCC, AG, insula, amygdala
Washington et al. (106)	ICA, Seed	B	Global/local		393	48	6	10.88	vACC/mPFC, dACC/mPFC, PCC, MTG, IPL
Weng et al. (107)	Seed	UC	Global		600	31	3	15	DMN (PCC/PRc, mPFC, AG)
Wiggins et al. (108)	SOM	UC	Global		600	80	15	15.3	DMN (PCC, PrC, Rsp, IPL, STG, mPFC, SFG, PHG)
You et al. (109)	SOM, GT	B	Global/local		308.4	31	10	11.2	Whole brain
Ypma et al. (110)	SEED	UC	Global	ABIDE	na	1,114	210	12–18	DMN *via* graph theoretical analysis based on prior meta-analytic findings. Sample reflects primary and ABIDE datasets only

*Inclusion required articles to directly compare individuals with ASD using resting-state fMRI. Authors examined the method of analysis used, the type of connectivity reported, and whether this connectivity was considered global or local as reported by the original authors. We were also interested in noting which studies utilized the ABIDE data set [Di Martino et al. (49)] and the sample represented in terms of sample size, mean age, and sex. We also include a report of brain regions examined (specifically for ROI Seed analyses) and networks explored. Superkar et al. (110) is also included as it is one of the first papers to examine the developmental changes in the DMN in a longitudinal study design*.

*AG, angular gyrus; ACC, anterior cingulate cortex; aIPS, anterior intraparietal sulcus; aMPFC, anterior medial prefrontal cortex; B, both; CEN, central executive network; CS, central sulcus; DMN, default mode network; DC, dorsal caudate; dlPFC, dorsolateral prefrontal cortex; GT, graph theory; ICA, independent component analysis; ICN, intrinsic connectivity networks; IFG, inferior frontal gyrus; IPC, intraparietal cortex; IPL, intraparietal lobule; LOS, lateral occipital sulcus; LPFC, lateral prefrontal cortex; mPFC, medial prefrontal cortex; MFG, medial frontal gyrus; MNS, mirror neuron system; MTG, medial temporal gyrus; na, not available; NACC, nucleus accumbens; OC, over-connectivity; OOC, occipital lobe; PHG, parahippocampal gyrus; PrC, precuneus; PCC, posterior cingulate cortex; PMC, premotor cortex; pSTS, posterior superior temporal sulcus; pTPJ, posterior temporal parietal junction; ReHo, regional homogeneity; RsC, retrosplenial cortex; Seed, region-of-interest seed-based analysis; SOM, self-organizing maps; SFG, superior frontal gyrus; STC, superior temporal cortex; STG, superior temporal gyrus; SN, salience network; S1, primary somatosensory cortex; TPJ, temporal parietal junction; ToM, Theory of Mind; vACC, ventral anterior cingulate cortex; UC, under-connectivity*.

*^a^Child, adolescent, and adult subgroups*.

## Terminology

Under-connectivity is often considered as a decrease in connectivity relative to a standard or normative comparison value. This can be a “global” decrease between different nodes of a network, like the posterior cingulate cortex (PCC) and the medial prefrontal cortex (mPFC) in the default mode network (DMN), or a “local” decrease in connectivity within a brain region, like changes within the PCC. For purposes of functional connectivity, under-connectivity means that the correlation between strength and timing of the BOLD signal in different voxels is decreased in affected subjects compared to unaffected comparisons subjects.

In contrast, OC (also termed hyperconnectivity, increased connectivity, or stronger connectivity) is reported when statistically significant correlations are present in the affected subject which are absent or less pronounced against the unaffected comparison. Thus, it may be possible that a brain region can be both under-connected with typically associated brain regions (53) while over-connected with non-traditional areas (63). Indeed, recent reports have identified more diffuse connectivity of brain networks in ASD, an example of OC in which brain regions are strongly correlated with ectopic regions outside of a traditionally defined circuit (64).

### Evidence of Under-Connectivity

The first studies of rs-fMRI in autism tended to support the under-connectivity theory (44, 46, 47, 56, 75, 107). Many early studies focused on connectivity of the DMN, a resting-state network (RSN) involved in introspective thought and self-reflection (15, 111). The DMN is active when a person is awake and alert but deactivates during cognitively demanding tasks or goal directed behavior (15, 111). Though DMN regional constituent has tended to vary study-by-study, the primary regions are the posterior cingulate cortex, medial prefrontal cortex, precuneus (PrC), and medial, lateral, and inferior parietal cortex (111). Research has focused on identifying DMN connectivity to explore the deficit in the ToM hypothesis (21).

One of the first studies examining the DMN did not have an explicit resting-state scan but rather compiled data from fixation blocks preceding a behavioral task and used a region-of-interest (ROI) seed-based analysis to examine FC (46). Although components of the DMN did not differ in ASD, anterior–posterior under-connectivity was found when compared to controls. Furthermore, connectivity patterns showed an emerging laterality with greater activation in the left, middle, and superior frontal gyrus (SFG) and supramarginal gyrus of ASD subjects and the right middle temporal gyrus of controls. These findings are complicated by the use of within-task fixation blocks as a substitute for an allocated resting-state scan which may require some care in separating from the task blocks around them (44, 46).

The first “true” rs-fMRI study was conducted by Kennedy and colleagues to compare intrinsic DMN connectivity in ASD and controls (48). The DMN was referred to as the “task-negative” network (TNN) because activity of core regions is attenuated during performance of a cognitive task. In contrast, the temporally anti-correlated “task-positive” network (TPN), including the dorsolateral prefrontal cortex (dlPFC), inferior parietal cortex, and supplementary motor area, is involved in outwardly focused cognitive tasks that require attention to the external environment (48, 111). The study demonstrated that TNN deactivation did not occur between resting-state and task-associated fMRI for ASD subjects (48).

A follow-up study by Kennedy and Courchesne used ROI seed-based analysis to examine resting-state FC of the TNN and TPN in ASD patients and controls (47). Since the TNN is considered important for socio-emotional behavior, both markedly affected in ASD, the authors predicted greater altered connectivity in the TNN compared to TPN, alternatively believed to support sustained attention and goal-directed behaviors that can be unimpaired in high-functioning ASD individuals. Results indicated under-connectivity within the TNN in ASD but no significant changes in the TPN (47). Notably, some research suggests that ASD individuals have deficits in sustained attention and goal-directed behavior (24, 33, 112), a discrepancy that may result from heterogeneity in the disorder. Further studies of DMN connectivity reported global under-connectivity (56, 76, 85, 100, 107, 108).

Some of these studies explored the relationship between DMN under-connectivity and symptom severity (56, 107). Assaf and colleagues demonstrated that under-connectivity was correlated with symptom severity on the ADOS social score, with higher scoring individuals (more severe symptomology) displaying a greater degree of DMN under-connectivity (56). Weng and colleagues repeated this finding with ROI seed-based analysis rather than independent component analysis (ICA) (107).

Recent research using the ABIDE data set delved into the origins of functional under-connectivity by investigating both inter- and intra-hemispheric connections. For purposes of the study, resting-state fMRI data were used to calculate global functional connectivity density, which was divided into ipsilateral and contralateral portions. Compared to controls, subjects with ASD exhibited inter- and intra-hemispheric under-connectivity in the posterior cingulate cortex, lingual/parahippocampal gyrus (PHG), and postcentral gyrus. When examining DMN regions, the study found global under-connectivity in the medial prefrontal cortex, posterior cingulate cortex, inferior parietal lobule, and sensorimotor regions. Based on their findings, Lee and colleagues postulated that under-connectivity of medial prefrontal cortex and posterior cingulate cortex contributes to social impairments (79).

DMN connectivity is best characterized by anterior–posterior under-connectivity (46, 48, 100, 108), specifically between frontal and parietal DMN nodes. This conclusion has been reached in numerous studies using ROI seed-based analysis, ICA, self-organizing maps (SOM), and voxel-wise whole brain analyses (Table 1 for details).

Research has expanded beyond DMN connectivity to examine other networks. Ebisch and colleagues examined connectivity of the insula cortex, a region considered important for awareness of “self” and others, and reported under-connectivity of the anterior and posterior insula (67). Another study reported under-connectivity in the insula (salience network) and amygdala (medial temporal lobe network) using both ICA and ROI seed-based analysis, also finding DMN under-connectivity (105, 110). Further studies showed reductions in limbic-related brain regions involved in social behavior, language, and communication (70).

Under-connectivity has also been indicated in voice perception (50) and language development (104) in ASD. Abrams and colleagues used the posterior superior temporal sulcus (pSTS) as the primary seed region to examine brain networks involved in the perception of human speech. They reported under-connectivity between the left pSTS and typical dopaminergic areas in ASD subjects, suggesting that ASD individuals experience a less pleasurable response to human voice processing (50). In addition, they reported decreased connectivity of the right pSTS with orbitofrontal and amygdala regions, areas that process speech prosody (rhythm and sound) (50). Alaerts and colleagues demonstrated that under-connectivity of the STS predicted the degree of emotional deficits in ASD (52).

A separate study by Verly and colleagues examined connectivity of networks underlying language development in an ASD group with clear comorbid language impairments (104). Subjects first performed a verb-generation task while in the fMRI to identify eight joint language components using ICA, which were then used as seed regions for analysis of the resting-state scan. Although connectivity between Wernicke’s and Broca’s language centers was preserved, under-connectivity was demonstrated between the right cerebellar region and supratentorial language areas in addition to under-connectivity of interhemispheric Broca’s area, as well as the dlPFC (104). These studies suggest under-connectivity may be responsible for communication deficits.

### Evidence for OC

A handful of rs-fMRI studies have reported OC in ASD using ROI seed-based analysis (60, 62–64, 87, 88) and Graph Theory and Network analysis (54, 97, 113). These studies have examined connectivity beyond the DMN, which may explain the findings of OC. Di Martino and colleagues first demonstrated OC of the striatum in ASD using a ROI seed-based analysis in school-age children (64). While OC was found between the striatum and regions previously implicated in ASD [right superior temporal gyrus and insular cortex], there was also extensive ectopic OC (64). Perhaps most notably, OC was reported between the striatum and pons, as well as between the pons and insular cortex (64). These findings indicate more expansive functional alterations in ASD than previously thought. A second study to examine striatal connectivity also found OC of frontostriatal connections and a trend toward OC of the right hemisphere (63).

Nebel and colleagues analyzed FC in the primary motor cortex (M1) and reported increased and less segregated connectivity of subregions of M1 in ASD (88). Laterality of OC was found for the right posterior temporoparietal junction, an area of the brain that integrates information from the external environment and the body, with the right ventral occipital-temporal cortex, confirmed using two analysis techniques (62). OC has also been reported between primary sensory and subcortical networks in the thalamus and basal ganglia. The degree of OC was correlated with symptom severity, suggesting that dysfunctional sensory connectivity may cause autistic behaviors (60).

Graph and network theory analysis have proved valuable for examining rs-fMRI in an unbiased, data-driven manner. Graph theory analyses contradict the majority of earlier literature and, depending on the study, have revealed increased connectivity or a combination of increased and decreased connectivity (54, 77, 96, 97, 113). The first study using this technique investigated the origin of ADHD symptoms often exhibited in ASDs (113). Cortical and subcortical areas displayed abnormal local degree centrality, indicating that the number of connections per node in ASD was increased relative to typically developing controls. Some regions of overlap existed between groups such as the PrC, while increased connectivity in bilateral temporolimbic regions was ASD-specific. Interestingly, when ASD individuals were categorized by presence or absence of ADHD symptoms, those with ADHD behavior shared ADHD-specific increased connectivity in the basal ganglia (113).

A separate study by Redcay and colleagues applied graph theory to examine FC of four distinct networks in ASD: DMN, cingulo-opercular, cerebellar, and frontoparietal networks (97). Minimal differences between ASD and non-ASD individuals were found in the adolescent males, except for greater betweenness centrality, an indicator of a node’s “centrality” in a network, in the ASD group for the right parietal region of the DMN (97). Anderson and colleagues reported increased connectivity in the DMN, with increased inter-network synchrony between the DMN and attentional networks in ASD (54).

Evidence for OC may also provide insight into the hypersensitivity to sensory stimuli and input seen in ASD. In a study by Cerliani and colleagues, functional connectivity between subcortical (basal ganglia and thalamus) and cortical RSNs was examined using ICA. Results indicated OC between primary sensory and subcortical regions, with overall connection strength positively correlated with ASD symptom severity (60).

### Evidence for Both Under- and Over-Connectivity

While a cursory glance would seem to suggest under-connectivity relative to OC, the majority of literature reports trends of both under- and over-connectivity in ASD, depending on whether one is examining local or global networks. The first study to demonstrate both types of connectivity, conducted by Monk and colleagues, evaluated DMN connectivity using the PCC component of the DMN as a single seed location in ASD adults (83). Findings indicated under-connectivity of the PCC and SFG in ASD subjects but OC between the PCC and bilateral temporal lobes as well as right-PHG. There was also a correlation between social functioning ADOS scores and PCC with temporal lobe connectivity, such that poorer social functioning correlated to weaker connectivity of PCC and SFG; alternatively, more severe restricted and repetitive behaviors were correlated with stronger connectivity of the PCC with the right-PHG (83).

Paakki and colleagues found both types of FC alterations in the DMN in children with ASD using regional homogeneity (ReHo), an alternative method that measures local synchronization of spontaneous fMRI (BOLD) signal activity of voxels within a given cluster (92). ReHo analysis of rs-fMRI data is very useful for examining local connectivity but cannot capture global or long range FC. The study demonstrated dominant right hemisphere alterations in ASD resting-state brain activity for areas previously exhibiting abnormal stimuli or task-related functionality in fMRI behavioral experiments and in DMN- associated regions (92). Decreased ReHo, equivalent to under-connectivity, was found in the right-superior temporal sulcus and right insula, areas that are associated with atypical sensory processing and problems with multisensory input integration (92).

Several groups have reported OC lateralizing to the right hemisphere (82, 99). Maximo and colleagues used a combination of ReHo and local density analysis from graph theory to demonstrate right hemisphere OC and left hemisphere under-connectivity in an early adolescent ASD population (82). Cardinale and colleagues came to a similar conclusion, reporting a rightward shift in increased intra-hemispheric connectivity and a decrease in left hemisphere connectivity using ICA methodology (59). The strong similarity in findings of these studies is likely attributed to similar age ranges represented across studies and use of whole brain analysis techniques. However, these studies are limited by use of ReHo analysis which does not allow for exploration of global connectivity. Now, we turn our attention to articles that use whole brain analytical techniques like graph theory to explore local and global connectivity alterations in ASD.

Early studies using SOM revealed a pattern of local OC and long distance under-connectivity in ASD, with reduced interhemispheric connectivity specific to the sensorimotor cortex, anterior insula, fusiform gyrus, superior temporal gyrus, and superior parietal lobule (55). Adolescents with ASD showed reduced short- and long-range connectivity within functional systems (measured by reduced functional integration), but stronger connectivity between functional systems (revealed by reduced functional segregation). This finding was particularly noticeable in default and higher order visual areas using graph theory analyses (98).

These findings support an emerging theory of less segregation within functional networks in ASD and more diffuse connectivity between networks. Increased diffuse connectivity provides a framework where reports of under- and over-connectivity can coexist because traditionally strong connections are weakened and new connections are present. Further support suggests a shift toward randomized or distributed network organization in ASD adults, evidenced by decreases in clustering coefficient and path length (73). There are reports of significantly altered organization of hub nodes in ASD adults, with a loss of “hubness” in select nodes [bilateral STS, right dorsal lateral prefrontal cortex (LPFC), and PrC] (73).

A recent publication used graph theory to address both inter- and intra-hemispheric FC in several rs-fMRI studies in adults with ASD using the ABIDE data set (71). They reported both over- and under-connectivity compared to controls, but found that FC patterns observed in typical controls were replaced by individual alterations in ASD patients (71). The diversity of literature results likely stem from the heterogeneous nature of ASD, whereby individual differences in FC organization, the idiosyncratic ASD connectivity maps, are themselves the best core feature of ASD (71). In summary, findings indicate global FC changes and suggest that difficulty in finding a predominant trend or type of change in ASD is due to the uniqueness of ASD for each individual.

With whole brain analysis suggesting a more diffuse pattern of connectivity for brain networks in ASD, we return to studies focused on the DMN to explore whether this holds true. Lynch and colleagues examined DMN connectivity in children with ASD and found OC of the PCC and retrosplenial cortex, primarily with medial and anterolateral cortices (81). OC of DMN-related circuits was positively correlated with social impairment severity. While under-connectivity was found between the PrC and visual cortex, basal ganglia, and locally with the posteriormedial cortex, the greater abundance of OC suggests that childhood ASD may be more characterized by OC (81).

A recent study conducted by Yerys and colleagues provides valuable insight into DMN rs-fMRI changes in non-medicated ASD children. As expected, functional under-connectivity was present between the medial prefrontal cortex (mPFC) and PCC regions of the DMN in ASD subjects (114). Furthermore, the degree of under-connectivity was associated with more severe social deficits. In addition, a combination of under- and over- connectivity between several DMN nodes and visual, subcortical motor, somatosensory, salience, ventral attention, and reward networks was found using seed-based analyses (114). When network analysis methods were utilized, the ASD group showed OC of the DMN with other networks compared to controls (114).

A complementary study by Washington and colleagues explored age-related changes in FC by dividing their sample into 6- to 9-year olds and 10- to 17-year olds (106). The study was designed to test two theories of ASD: first, that ASD is characterized by deficits in ToM and, second, that ASD is marked by global under-connectivity and local OC (106). Using ICA and ROI seed-based analysis, they found under-connectivity between DMN nodes but local OC within DMN brain regions as well as visual and motor RSNs. This between-node connectivity in the DMN increased with age in the typically developing controls but was absent in the ASD children, lending support to the “developmental disconnection model” of ASD (106). The findings from this study differ from previous reports of under-connectivity in the DMN, but raise important questions regarding connectivity changes with age. Changes may be more apparent at select ages though adjustment is needed to account for changes as a result-of-life experience.

Recent research by Olivito and colleagues examined FC between the cerebellum and ToM/DMN-associated regions. Previous studies indicated cerebellar influence on cerebral cortex activity through cerebello–thalamo–cortical circuits, with dysfunction possibly causing ASD behaviors. In this study, resting-state under-connectivity was found between the left cerebellar DN and cerebral regions, most notably the right-sided medial frontal, posterior temporal, and posterior parietal regions. Secondary analysis of the cerebellum demonstrated OC between the left DN and supramodal cerebellar lobules linked to the DMN (91).

Doyle-Thomas and colleagues further explored DMN connectivity in children with ASD and demonstrated both over- and under-connectivity of the PCC depending on the DMN region (66). The PCC of ASD subjects was over-connected with regions not associated with the DMN. PCC connectivity with the mPFC increased with age in controls [previously shown by Ref. (101)], but this connection was decreased in ASD. Mixed patterns of connectivity in the DMN continue to be reported (38) as well as OC between regions of the DMN and the MNS. An analysis restricted to individuals with the most severe ASD symptomatology exclusively showed OC between the DMN and MNS (38).

These findings again emphasize the need to address both age and severity when forming conclusions about FC in ASD. Another important consideration when interpreting results and comparing conclusions is the individual’s mental state during the scan (115). It is challenging to control an individual’s mental state during a resting-state scan and different instructions (i.e., keeping eyes open or closed) can cause significant variances in the data (115). This is exacerbated by an individual’s attenuation to an external or internal stimulus, and our inability to measure the degree of “introspection” or “mind wandering” when examining a brain network attributed to these mental behaviors. It is, thus, necessary to attempt to manipulate mental states in ASD rs-fMRI in order to explore differences in FC between mental states and draw comparisons with controls. Barttfeld and colleagues were the first to study differences in FC across mental states in ASD adults (57). They compared the large-scale functional connectivity of six functional networks (frontoparietal, cingulo-opercular, DMN, occipital, sensorimotor, and cerebellar networks) across three different cognitive states: an interoceptive condition (attend to an internal cue such as breathing), an exteroceptive condition (count the number of sounds), and an eyes-closed “resting state” condition. Under-connectivity was observed in the resting-state and exteroceptive conditions, while OC was observed during the introspective attentional task. These changes were more pronounced between states for ASD subjects than controls and were best characterized by the FC of the anterior insula and anterior cingulate cortex (57). A similar finding was reported by You and colleagues (109), who found more diffuse connectivity during performance of a sustained attention task in children with ASD compared to their resting-state FC. Resting-state connectivity was also reduced in ASD children and became more diffuse during task performance compared to typically developing children where FC became more focal. The flexibility of the brain in different states has shown it to be less differentiated in ASD compared to TDC in the DMN, the salience network (SN) comprised of the right frontal insula and anterior cingulate cortex, and the central executive network comprised of the right-dorsal LPFC and right PCC (103).

## The Question of Connectivity Revisited

Even upon reviewing the rs-fMRI literature, it is challenging to draw direct conclusions about FC in ASD. Strides have been made to introduce data-sharing initiatives, such as the ABIDE network, a large-scale open access repository for ASD neuroimaging data that currently has over 1,112 resting-state data sets from 17 data collection sites (49). Fifteen reviewed papers utilized this data set (in addition to the original publication by Di Martino et al. (49), for a total of 16 papers) to investigate resting-state connectivity and provide further insight (49–51, 60, 61, 71, 72, 79, 80, 88–90, 94, 96, 113). Many of these studies found instances of both over- and under-connectivity in ASD (49, 51, 61, 71, 72, 88, 90), supporting the idea of more diffuse connectivity in ASD.

The lack of consensus regarding connectivity has not stopped researchers from exploring the use of altered connectivity as a biomarker for ASD diagnosis (55, 57, 65, 72, 76, 85, 89, 94, 95, 103). Several groups have tested classifier models, from machine learning (94) and support vector machine (57), to leave-one-out classifiers (53, 89) and probabilistic neural networks (72). There have been varying degrees of predictive success, none exceeding 90% accuracy, when using one data set to inform the model to make predictions on a novel data set. Unfortunately, many of these classifiers currently lack retest reliability when applied to novel ASD data sets (53, 89, 94, 95).

## Limitations and Considerations for rs-fMRI

Care should be taken when reviewing ASD rs-fMRI literature to address study parameters that may limit inferences made about large-scale ASD populations. These include the diversity of the subject pool (age and sex representation), the design of the resting-state scan, and the preprocessing and methodology of analysis. Total sample size is also an important variable with a range of 24–115 subjects in the literature [1,112 subjects when we consider studies utilizing the ABIDE data set; (49)]. The pattern of increasing sample size suggests more reliability in the reported results, given that statistical significance should have sufficient power with greater sample size. However, there are important non-statistical considerations such as relative gaps in age and sex representation in studied sample populations.

Sex representation in the literature is remarkably unbalanced, with females representing only 10% of the mean sample size per study. Of the studies reviewed here, 18 excluded females altogether. In our research, no studies were found that equally represented males and females when assessing resting-state FC. Although ASD is 4.5 times more likely to be diagnosed in males, the relative absence of females limits our understanding of ASD in females, who are still diagnosed, albeit at much lower rates. Attempting to remedy this disparity by using the readily available ABIDE data set to explore female-specific changes in connectivity in ASD would be a great contribution to the literature.

Age is also an important consideration when drawing conclusions about ASD connectivity changes, shown in Figure 1A. The current mean age for the studies reviewed here was 17.26 years with a range of 2.42–35.5 years. This makes broader inferences more difficult because of the brain’s plasticity and developmental potential through adolescence, such that reports of connectivity at an older age may not represent connectivity at a younger age. This issue has begun to be addressed in the DMN in typically developing children (101, 116, 117), and through comparisons of changes in connectivity with age in ASD (51, 58, 80, 90, 93, 106). These developmentally focused age comparison studies provide a clearer picture of connectivity alterations in ASD individuals. It would, however, be beneficial to gather more information on children in the 3- to 8-year age bracket as they are not well-represented at this time. Admittedly, this age range is challenging given young children’s difficulties remaining still during the scan, resulting in greater motion artifact.

This age range represents a pivotal time as children are first being diagnosed, interventions and treatments are being prescribed, and the brain is undergoing major developmental changes. It is worth noting that most of the studies thus far have examined ASD in older individuals who were diagnosed at a younger age and have already received years of treatment, education, and therapy. Only one study has looked exclusively at rs-fMRI connectivity in naturally sleeping toddlers, where the mean age was 2.42 years (65). Last, one should carefully consider the ASD subgroup tested for a study before making larger inferences as studies generally include higher functioning ASD individuals with relatively normal IQ scores.

It is still unclear when differences in connectivity in ASD first emerge, to what degree experience either exaggerates or minimizes these differences and what biological causes may drive these differences. It is also unclear whether altered connectivity is a result of a failure to generate connections, inappropriate pruning, or creation of new connections.

## Parametric, Preprocessing, and Methodological Considerations

The design of the resting-state scan itself may contribute to variability in data findings. Scan length varies from 283 s to 600 s with a mean scan length of 417.6 s, or about 7 min. The mode, however, appears to be 360 s or 6 min. Over the time course of rs-fMRI neuroimaging, the length of scan time seems to have decreased, although evidence suggests that better results and more accurate classification are found using longer scan times (89). Two further issues with scan parameters are variability in the number of volumes collected by study and differing instructions given to subjects. While the majority of studies instruct participants to fixate on a plus sign with eyes open and subsequently let their minds wander, others instruct participants to keep their eyes closed. What appears to be a minor difference in instructions can result in different mental states and ultimately different outcome measures of functional connectivity. Standardizing the format of the rs-fMRI scan would greatly assist in drawing more meaningful comparisons between studies, especially given evidence that changes in mental state affect FC (57, 103, 109). Last, it is important to note that some earlier studies attempted to examine FC using within-block residuals with the task signal regressed out (65, 99), in addition to using short 15–24 s fixation blocks (46, 48, 85) as the resting-state analog. One should be cautious about drawing conclusions regarding resting-state functional connectivity from studies lacking a true resting-state scan.

Data preprocessing to control for movement and physiological noise can profoundly impact the reported results (78, 86, 102, 118, 119). To reduce its impact in fMRI (119) studies, it is uniformly applied to subjects. However, its ability to provide more accurate analysis of imaging data compensates for its inherent shortcomings. The method of analysis, however, is the most likely cause of ongoing discrepancies in the literature.

While each methodology has strengths and weaknesses, some hold greater representation in the literature. ROI seed-based analysis is the predominant choice for examining functional connectivity in resting-state analysis, with just over half of all current studies in the literature utilizing this technique (see Table 1). Seed based analysis, wherein the researcher selects a ROI and specifies the coordinates that comprise the ROI, is primarily a hypothesis-driven method of analysis. It has been particularly useful for examining the DMN in ASD, where the ROIs are pre-specified using previous publications on DMN regions in non-ASD individuals. In addition to local connectivity, it can be used to look at global and interhemispheric connections. A significant downside of this method is its inability to inspect the FC of regions connected to, but outside of, the seed region. This limits findings to the preselected ROIs, potentially causing significant alterations to be overlooked.

Independent component analysis is the second most common analysis type in the literature and utilizes a data-driven approach (56, 58–60, 90, 95, 100, 101, 120). For this method, a mask is laid on the data of defined brain regions, and a set number of components are computed through spectral decomposition of the correlation matrix. With this approach, some components are deemed “noise” while others are labeled as “signal” components and represent brain areas or networks. Components are designed to represent accepted networks when multiple brain regions associated with a network contribute to the same component.

One drawback to ICA analysis is that a single brain region can be found in multiple components and frequently the components themselves are not orthogonal to each other, leading to proposed network correlations that are instead an effect of mathematical data handling. Another issue with ICA is that it must be done independently for ASD and non-ASD control data, making comparison between results more challenging, though not impossible. ICA is particularly useful for whole brain connectivity analysis and, unlike ROI seed-based analysis, is not limited to predefined brain networks. However, the use of predefined networks is used to categorize components from the analysis. ICA remains a strong analysis tool in the literature for global connectivity questions.

Regional homogeneity is a very useful technique for exploring local connectivity within a brain region by exploring the local correlation synchrony of adjacent voxels. A significant drawback is that ReHo analysis cannot extend past the local level and thus has only been used in a handful of studies (49, 51, 82, 92). Whole brain voxel-wise analysis became more popular, using a similar approach to ReHo but allowing whole brain analysis of voxel correlations. This technique has been used by several studies to explore FC in ASD (70–72, 85) and is able to capture both global and local analysis of FC. Voxel-wise analysis is a data-driven approach as well, but can be used in conjunction with seed-based analysis, in which case the data is first analyzed using voxel-wise and then specific ROIs are explored. A similar analysis method is the SOM (53, 55, 75, 103, 108, 109). SOM analysis is a dimension reduction and data visualization technique that organizes data that are alike into nodes on a lower dimensional display, such that nodes close together in a matrix represent neural networks. One can then identify clusters containing hubs of pre-identified networks and use them to make comparisons between groups.

A recent increase in the use of graph theory and network analysis of FC in rs-fMRI has emerged in the literature (82, 121, 122). Graph theory and network analysis techniques are extremely useful in studying ASD because they can examine both local and global changes in network connectivity and strength. They are also capable of highlighting connectivity changes in prototypically recognized networks like the DMN. Graph theory is a powerful tool for elucidating ASD brain connectivity alterations and the mechanisms underlying key features of the disorder. The application of graph theory and connectomics may help resolve discrepancies in the literature.

Ultimately, all types of analyses have their limitations and advantages. The use of multiple analysis types in a single study would provide a greater description of the altered connectivity than one analysis method alone. Such multi-method analyses have been done with some contradictory findings reported.

## Propagation of Intrinsic Activity

Resting-state fMRI analysis relies on the assumption that intrinsic brain activity is perfectly synchronous within RSNs. In a recent study, Mitra and colleagues found reproducible lag times in the propagation of intrinsic brain activity within rs-fMRI data (123). Most notably, the frontopolar cortex, occipital cortex, and putamen were found to have statistically significant lag time differences when comparing controls to ASD subjects. The frontopolar cortex was late with respect to the rest of the brain in controls but not ASD subjects, the occipital cortex was early in controls but late in ASD subjects, and putamen lag time was near 0 for controls and significantly early in ASD subjects. Furthermore, frontopolar cortex lag time was negatively correlated with attention problem scores, such that decreased lag time was associated with more severe attention deficits. Stronger putamen propagation (inferred from early propagation with respect to the rest of the brain) was positively correlated with restricted and repetitive behaviors. Interestingly, functional connectivity analysis from the same rs-fMRI data found no significant differences (123). This raises new questions about conventional rs-fMRI use and a potential limitation of current analyses.

## Limitations of Classifier Models

Several studies have attempted to use FC differences between ASD and non-ASD groups to design a classifier model capable of predicting ASD in a given individual. The hope is that hallmark connectivity patterns could inform a strong classifier model to predict ASD with the potential for biomarker utility. A classifier, built using one data set, is then tested for accuracy by applying the built-in parameters to novel data. The classifier is thus trained by using machine learning to differentiate group patterns and apply the resulting model to new data. The accuracy is determined by the number of “false positive” and “false negative” hits that occur. Researchers have attempted to use the pattern of under-connectivity (55, 65, 76, 85) and other forms of altered connectivity (49, 72) as autism biomarkers to inform classifier models to predict ASD diagnosis. While classifiers perform with modest to conservatively good accuracy rates, they have trouble replicating to new data sets (72, 76, 95).

Perhaps more problematic, the majority of these studies use data from ASD individuals in late adolescence to early adulthood. The drawback is that connectivity changes occur throughout development and early diagnosis is crucial for improved prognosis, treatment, and outcome. For this reason, designing classifier models using adult brain connectivity would be inefficient at predicting ASD patterns of connectivity in a childhood data set. The Dinstein et al. study is unique as it is the only study reviewed in this paper to examine FC in toddlers (65). As mentioned previously, researchers found that ASD toddlers had weaker interhemispheric connectivity in putative language areas compared to typically developing toddlers and toddlers with language delays (65). Attempts to use these results as a classifier yielded only 72% sensitivity and 84% specificity.

## Conclusion and Future Directions

While hallmark connectivity patterns are still unclear, evidence suggests that ASD is most likely characterized by instances of both under- and over-connectivity. Evidence of a more diffuse pattern of connectivity in autism is supported by the literature. More research is necessary to determine characteristic FC alterations in ASD, the time at which alterations occur, and whether these changes occur on a spectrum from ASD to “typically developing” individuals.

Large-scale data-sharing initiatives like the ABIDE network and NIH initiative for a national database of autism research will help advance research by making data easily accessible for analysis. Due to the heterogeneous nature of autism, it is perhaps best to begin using more data drive approaches to understand the diversity of brain connectivity. Such an approach would allow researchers to analyze brain scans without predefined DSM-V diagnosis and allow for the data to form natural clusters of similarities and drive differences between ASD and non-ASD individuals. Results could then inform more natural data-driven categorization models of individuals based on their connectivity. While use of rs-fMRI as a diagnostic tool is an interesting idea, it is still relatively premature in its practical application and will benefit greatly from a clearer understanding of functional connectivity differences between experimental and control groups. For the continued purposes of clarity, however, greater precision may be required to avoid the use of less focused terms such as “under-connectivity” and “over-connectivity.”

The continued interplay of cognitive theories and neuroimaging results has an important role in advancing our knowledge of ASD. Looking toward the future of rs-fMRI research, it is important to focus on characterization of ASD in the more juvenile brain, the female brain, and perhaps investigation of the impact of early treatments on the developing brain’s plasticity.

## Author Contributions

JH, LD, and JDVH contributed to the conception, research, and preparation of the manuscript. ZJ, CT, and AI provided critical intellectual input on the narrative and conclusions of the manuscript. ZJ also contributed detailed author proof edits on the final published version of the manuscript. JH and LD serve as joint first authors of this work. All authors provide approval for publication of the content.

## Conflict of Interest Statement

The authors declare that the research was conducted in the absence of any commercial or financial relationships that could be construed as a potential conflict of interest.
