# Corrigendum: Resting-State Functional Connectivity in Autism Spectrum Disorders: A Review

**DOI:** 10.3389/fpsyt.2018.00268

**Published:** 2018-06-22

**Authors:** Jocelyn V. Hull, Lisa B. Dokovna, Zachary J. Jacokes, Carinna M. Torgerson, Andrei Irimia, John Darrell Van Horn, the GENDAAR Research Consortium

**Affiliations:** Laboratory of Neuro Imaging (LONI), The Institute for Neuroimaging and Informatics (INI), Keck School of Medicine of USC, University of Southern California, Los Angeles, CA, United States

**Keywords:** resting state, fMRI, functional connectivity, autism spectrum disorder, developmental brain imaging, neural networks

Due to an unfortunate oversight at the time of final submission of our original article, Ms. Lisa Beth Dokovna, who made important contributions to this article was not included as joint first-author on the authorship list. Rather, she was included in the acknowledgments section. The authors apologize for this error which was an honest mistake and one that they would like to rectify. This correction, however, does not change the scientific conclusions of the original article in any way.

The updated Author Contribution Statement appears below. We also wish to mention the financial support in our Funding Statement. Finally, please also find a corrected Acknowledgments section below to replace the one in place presently.

The original article has been updated.

## Author contributions

JH, LD, and JDVH contributed to the conception, research, and preparation of the manuscript. ZJ, CT, and AI provided critical intellectual input on the narrative and conclusions of the manuscript. ZJ also contributed detailed author proof edits on the final published version of the manuscript. JH and LD serve as joint first authors of this work. All authors provide approval for publication of the content.

### Conflict of interest statement

The authors declare that the research was conducted in the absence of any commercial or financial relationships that could be construed as a potential conflict of interest.

